# Supplementary material for: Convective heat transfer of the Taylor flow in a two-dimensional piston pump
Source: PLoS One. 2022 Oct 13;17(10):e0275897. doi: 10.1371/journal.pone.0275897 (PMC9560506; doi:10.1371/journal.pone.0275897)
Supplement: S1 Table — (DOCX) [file pone.0275897.s001.docx]

| **S1 Table. The experimental and simulation values with calculated results at 1000 rpm.** | | | | | | | | | | |
| --- | --- | --- | --- | --- | --- | --- | --- | --- | --- | --- |
| ***t*** | $\text{T}_{\text{oil}}$ | $\text{T}_{\text{No.2}}$ | $\text{T}_{\text{No.3}}$ | $\text{T}_{\text{No.4}}$ | $\text{T}_{\text{r2}}$ | $\text{T}_{\text{r2}\text{s}}$ | $\text{R}_{\text{e}}$ | $\text{T}_{\text{a}}$ | $\text{h}_{\text{1}}$ | $\text{N}_{\text{u}\text{1}}$ |
| 0 | 28.3 | 28.0 | 28.0 | 28.0 | 28.00 | 28.09 | 234.36 | 1373.15 | 318.79 | 14.51 |
| 10 | 28.4 | 28.1 | 28.1 | 28.1 | 28.10 | 28.19 | 235.55 | 1387.14 | 328.95 | 14.97 |
| 20 | 28.5 | 28.2 | 28.2 | 28.2 | 28.20 | 28.29 | 236.74 | 1401.10 | 338.99 | 15.43 |
| 30 | 28.6 | 28.3 | 28.3 | 28.3 | 28.30 | 28.39 | 237.91 | 1415.03 | 348.86 | 15.88 |
| 40 | 28.7 | 28.4 | 28.4 | 28.4 | 28.40 | 28.50 | 239.07 | 1428.92 | 358.56 | 16.32 |
| 50 | 28.8 | 28.5 | 28.5 | 28.5 | 28.50 | 28.60 | 240.23 | 1442.76 | 368.04 | 16.76 |
| 60 | 28.9 | 28.6 | 28.6 | 28.6 | 28.60 | 28.70 | 241.37 | 1456.55 | 377.30 | 17.18 |
| 70 | 29.0 | 28.7 | 28.7 | 28.7 | 28.70 | 28.77 | 242.51 | 1470.27 | 386.31 | 17.59 |
| 80 | 29.0 | 28.8 | 28.8 | 28.8 | 28.80 | 28.85 | 243.63 | 1483.93 | 395.06 | 17.99 |
| 90 | 29.1 | 28.9 | 28.9 | 28.9 | 28.90 | 28.93 | 244.75 | 1497.52 | 403.53 | 18.38 |
| 100 | 29.2 | 29.0 | 29.0 | 29.0 | 29.00 | 29.02 | 245.85 | 1511.02 | 411.72 | 18.75 |
| 110 | 29.3 | 29.0 | 29.0 | 29.0 | 29.00 | 29.11 | 246.94 | 1524.45 | 419.62 | 19.11 |
| 120 | 29.4 | 29.1 | 29.1 | 29.1 | 29.10 | 29.18 | 248.01 | 1537.78 | 427.24 | 19.46 |
| 130 | 29.4 | 29.2 | 29.2 | 29.2 | 29.20 | 29.25 | 249.08 | 1551.03 | 434.56 | 19.79 |
| 140 | 29.5 | 29.3 | 29.3 | 29.3 | 29.30 | 29.33 | 250.13 | 1564.17 | 441.60 | 20.11 |
| 150 | 29.6 | 29.4 | 29.4 | 29.4 | 29.40 | 29.42 | 251.17 | 1577.22 | 448.37 | 20.42 |
| 160 | 29.7 | 29.5 | 29.4 | 29.5 | 29.47 | 29.52 | 252.20 | 1590.15 | 454.86 | 20.72 |
| 170 | 29.8 | 29.6 | 29.5 | 29.5 | 29.53 | 29.59 | 253.22 | 1602.98 | 461.10 | 21.00 |
| 180 | 29.8 | 29.6 | 29.6 | 29.6 | 29.60 | 29.65 | 254.22 | 1615.69 | 467.10 | 21.28 |
| 190 | 29.9 | 29.7 | 29.7 | 29.7 | 29.70 | 29.74 | 255.21 | 1628.29 | 472.87 | 21.54 |
| 200 | 30.0 | 29.8 | 29.7 | 29.8 | 29.77 | 29.82 | 256.19 | 1640.77 | 478.43 | 21.80 |
| 210 | 30.1 | 29.8 | 29.8 | 29.8 | 29.80 | 29.92 | 257.15 | 1653.14 | 483.80 | 22.04 |
| 220 | 30.2 | 30.0 | 30.0 | 30.0 | 30.00 | 30.01 | 258.10 | 1665.37 | 488.99 | 22.28 |
| 230 | 30.3 | 30.0 | 30.0 | 30.0 | 30.00 | 30.08 | 259.04 | 1677.49 | 494.02 | 22.51 |
| 240 | 30.3 | 30.1 | 30.1 | 30.1 | 30.10 | 30.15 | 259.96 | 1689.48 | 498.92 | 22.73 |
| 250 | 30.4 | 30.2 | 30.2 | 30.2 | 30.20 | 30.23 | 260.87 | 1701.35 | 503.69 | 22.95 |
| 260 | 30.5 | 30.3 | 30.2 | 30.2 | 30.23 | 30.29 | 261.77 | 1713.09 | 508.37 | 23.17 |
| 270 | 30.5 | 30.3 | 30.3 | 30.3 | 30.30 | 30.36 | 262.66 | 1724.71 | 512.95 | 23.37 |
| 280 | 30.6 | 30.4 | 30.4 | 30.4 | 30.40 | 30.41 | 263.53 | 1736.21 | 517.47 | 23.58 |
| 290 | 30.6 | 30.4 | 30.4 | 30.4 | 30.40 | 30.46 | 264.39 | 1747.58 | 521.93 | 23.79 |
| 300 | 30.7 | 30.5 | 30.4 | 30.5 | 30.47 | 30.51 | 265.24 | 1758.83 | 526.35 | 23.99 |
| 310 | 30.7 | 30.5 | 30.5 | 30.5 | 30.50 | 30.57 | 266.08 | 1769.97 | 530.74 | 24.19 |
| 320 | 30.8 | 30.6 | 30.6 | 30.6 | 30.60 | 30.61 | 266.91 | 1780.99 | 535.11 | 24.39 |
| 330 | 30.8 | 30.6 | 30.6 | 30.6 | 30.60 | 30.67 | 267.72 | 1791.90 | 539.48 | 24.59 |
| 340 | 30.9 | 30.7 | 30.7 | 30.7 | 30.70 | 30.74 | 268.53 | 1802.70 | 543.84 | 24.79 |
| 350 | 31.0 | 30.8 | 30.8 | 30.8 | 30.80 | 30.80 | 269.32 | 1813.39 | 548.20 | 24.99 |
| 360 | 31.0 | 30.9 | 30.9 | 30.9 | 30.90 | 30.86 | 270.11 | 1823.99 | 552.56 | 25.19 |
| 370 | 31.1 | 30.9 | 30.9 | 30.9 | 30.90 | 30.94 | 270.89 | 1834.49 | 556.93 | 25.39 |
| 380 | 31.2 | 31.0 | 31.0 | 31.0 | 31.00 | 31.02 | 271.65 | 1844.91 | 561.30 | 25.59 |
| 390 | 31.3 | 31.0 | 31.0 | 31.0 | 31.00 | 31.09 | 272.41 | 1855.24 | 565.66 | 25.79 |
| 400 | 31.3 | 31.1 | 31.1 | 31.1 | 31.10 | 31.12 | 273.17 | 1865.50 | 570.01 | 25.98 |
| 410 | 31.3 | 31.1 | 31.1 | 31.1 | 31.10 | 31.14 | 273.91 | 1875.69 | 574.33 | 26.18 |
| 420 | 31.3 | 31.2 | 31.2 | 31.2 | 31.20 | 31.18 | 274.65 | 1885.83 | 578.61 | 26.38 |
| 430 | 31.4 | 31.2 | 31.2 | 31.2 | 31.20 | 31.25 | 275.38 | 1895.91 | 582.82 | 26.57 |
| 440 | 31.5 | 31.3 | 31.3 | 31.3 | 31.30 | 31.30 | 276.11 | 1905.95 | 586.95 | 26.76 |
