# Supplementary material for: Convective heat transfer of the Taylor flow in a two-dimensional piston pump
Source: PLoS One. 2022 Oct 13;17(10):e0275897. doi: 10.1371/journal.pone.0275897 (PMC9560506; doi:10.1371/journal.pone.0275897)
Supplement: S2 Table — (DOCX) [file pone.0275897.s002.docx]

| **S2 Table. The experimental and simulation values with calculated results at 1500 rpm.** | | | | | | | | | | |
| --- | --- | --- | --- | --- | --- | --- | --- | --- | --- | --- |
| ***t*** | $\text{T}_{\text{oil}}$ | $\text{T}_{\text{No.2}}$ | $\text{T}_{\text{No.3}}$ | $\text{T}_{\text{No.4}}$ | $\text{T}_{\text{r2}}$ | $\text{T}_{\text{r2}\text{s}}$ | $\text{R}_{\text{e}}$ | $\text{T}_{\text{a}}$ | $\text{h}_{\text{1}}$ | $\text{N}_{\text{u}\text{1}}$ |
| 0 | 27.1 | 26.7 | 26.7 | 26.6 | 26.67 | 26.23 | 332.09 | 2757.09 | 463.44 | 21.08 |
| 10 | 27.6 | 27.2 | 27.0 | 27.1 | 27.10 | 26.90 | 338.52 | 2864.82 | 467.62 | 21.28 |
| 20 | 28.0 | 27.5 | 27.5 | 27.5 | 27.50 | 27.28 | 344.88 | 2973.52 | 471.94 | 21.48 |
| 30 | 28.4 | 27.9 | 27.9 | 27.8 | 27.87 | 27.66 | 351.17 | 3083.09 | 476.37 | 21.68 |
| 40 | 28.8 | 28.2 | 28.2 | 28.3 | 28.23 | 28.05 | 357.40 | 3193.41 | 480.93 | 21.90 |
| 50 | 29.1 | 28.6 | 28.6 | 28.7 | 28.63 | 28.41 | 363.56 | 3304.37 | 485.59 | 22.11 |
| 60 | 29.5 | 29.0 | 29.0 | 28.9 | 28.97 | 28.76 | 369.64 | 3415.87 | 490.38 | 22.33 |
| 70 | 29.8 | 29.3 | 29.3 | 29.3 | 29.30 | 29.11 | 375.65 | 3527.79 | 495.27 | 22.56 |
| 80 | 30.1 | 29.6 | 29.6 | 29.7 | 29.63 | 29.43 | 381.58 | 3640.03 | 500.27 | 22.79 |
| 90 | 30.4 | 30.0 | 30.0 | 29.9 | 29.97 | 29.75 | 387.43 | 3752.48 | 505.38 | 23.03 |
| 100 | 30.7 | 30.2 | 30.2 | 30.3 | 30.23 | 30.05 | 393.19 | 3865.06 | 510.59 | 23.27 |
| 110 | 31.0 | 30.5 | 30.5 | 30.4 | 30.47 | 30.35 | 398.88 | 3977.64 | 515.92 | 23.51 |
| 120 | 31.2 | 30.7 | 30.7 | 30.8 | 30.73 | 30.62 | 404.48 | 4090.16 | 521.35 | 23.76 |
| 130 | 31.4 | 31.0 | 31.0 | 31.0 | 31.00 | 30.86 | 410.00 | 4202.50 | 526.89 | 24.02 |
| 140 | 31.7 | 31.3 | 31.3 | 31.2 | 31.27 | 31.11 | 415.43 | 4314.58 | 532.54 | 24.28 |
| 150 | 32.0 | 31.6 | 31.6 | 31.5 | 31.57 | 31.38 | 420.78 | 4426.33 | 538.30 | 24.55 |
| 160 | 32.3 | 31.9 | 31.8 | 31.8 | 31.83 | 31.67 | 426.04 | 4537.65 | 544.17 | 24.82 |
| 170 | 32.5 | 32.1 | 32.1 | 32.1 | 32.10 | 31.93 | 431.21 | 4648.47 | 550.15 | 25.09 |
| 180 | 32.7 | 32.3 | 32.3 | 32.3 | 32.30 | 32.16 | 436.29 | 4758.72 | 556.25 | 25.37 |
| 190 | 33.0 | 32.6 | 32.5 | 32.5 | 32.53 | 32.41 | 441.29 | 4868.34 | 562.46 | 25.66 |
| 200 | 33.1 | 32.8 | 32.7 | 32.7 | 32.73 | 32.62 | 446.20 | 4977.25 | 568.78 | 25.95 |
| 210 | 33.4 | 33.0 | 32.9 | 32.9 | 32.93 | 32.84 | 451.02 | 5085.41 | 575.23 | 26.25 |
| 220 | 33.6 | 33.2 | 33.1 | 33.1 | 33.13 | 33.07 | 455.75 | 5192.75 | 581.79 | 26.55 |
| 230 | 33.8 | 33.4 | 33.4 | 33.4 | 33.40 | 33.29 | 460.40 | 5299.23 | 588.47 | 26.86 |
| 240 | 33.9 | 33.6 | 33.6 | 33.6 | 33.60 | 33.46 | 464.97 | 5404.81 | 595.27 | 27.17 |
| 250 | 34.1 | 33.8 | 33.8 | 33.8 | 33.80 | 33.64 | 469.44 | 5509.45 | 602.20 | 27.49 |
| 260 | 34.3 | 34.0 | 33.9 | 33.9 | 33.93 | 33.83 | 473.84 | 5613.11 | 609.25 | 27.81 |
| 270 | 34.5 | 34.1 | 34.1 | 34.1 | 34.10 | 34.02 | 478.15 | 5715.76 | 616.43 | 28.14 |
| 280 | 34.6 | 34.3 | 34.3 | 34.3 | 34.30 | 34.19 | 482.38 | 5817.38 | 623.74 | 28.48 |
| 290 | 34.8 | 34.5 | 34.5 | 34.5 | 34.50 | 34.35 | 486.54 | 5917.95 | 631.18 | 28.82 |
| 300 | 35.0 | 34.7 | 34.6 | 34.7 | 34.67 | 34.54 | 490.61 | 6017.47 | 638.75 | 29.17 |
| 310 | 35.2 | 34.8 | 34.8 | 34.8 | 34.80 | 34.73 | 494.61 | 6115.91 | 646.46 | 29.53 |
| 320 | 35.3 | 35.0 | 35.0 | 35.0 | 35.00 | 34.89 | 498.53 | 6213.27 | 654.30 | 29.89 |
| 330 | 35.5 | 35.2 | 35.1 | 35.2 | 35.17 | 35.06 | 502.38 | 6309.56 | 662.27 | 30.25 |
| 340 | 35.7 | 35.3 | 35.3 | 35.3 | 35.30 | 35.25 | 506.15 | 6404.78 | 670.38 | 30.62 |
| 350 | 35.7 | 35.4 | 35.4 | 35.4 | 35.40 | 35.37 | 509.86 | 6498.95 | 678.62 | 31.00 |
| 360 | 35.9 | 35.6 | 35.5 | 35.5 | 35.53 | 35.50 | 513.50 | 6592.07 | 687.00 | 31.39 |
| 370 | 36.0 | 35.7 | 35.7 | 35.7 | 35.70 | 35.64 | 517.07 | 6684.16 | 695.52 | 31.78 |
| 380 | 36.2 | 35.9 | 35.9 | 35.9 | 35.90 | 35.79 | 520.59 | 6775.26 | 704.17 | 32.18 |
| 390 | 36.3 | 36.0 | 36.0 | 36.0 | 36.00 | 35.93 | 524.04 | 6865.39 | 712.96 | 32.58 |
| 400 | 36.5 | 36.2 | 36.2 | 36.2 | 36.20 | 36.08 | 527.43 | 6954.57 | 721.89 | 32.99 |
| 410 | 36.6 | 36.4 | 36.3 | 36.3 | 36.33 | 36.22 | 530.77 | 7042.86 | 730.94 | 33.41 |
| 420 | 36.8 | 36.5 | 36.5 | 36.5 | 36.50 | 36.38 | 534.05 | 7130.29 | 740.13 | 33.83 |
| 430 | 37.0 | 36.7 | 36.6 | 36.7 | 36.67 | 36.55 | 537.29 | 7216.91 | 749.45 | 34.26 |
| 440 | 37.1 | 36.8 | 36.8 | 36.8 | 36.80 | 36.70 | 540.47 | 7302.77 | 758.89 | 34.69 |
| 450 | 37.3 | 37.0 | 36.9 | 37.0 | 36.97 | 36.86 | 543.61 | 7387.91 | 768.46 | 35.13 |
| 460 | 37.4 | 37.1 | 37.0 | 37.1 | 37.07 | 37.01 | 546.71 | 7472.41 | 778.14 | 35.58 |
| 470 | 37.5 | 37.2 | 37.2 | 37.2 | 37.20 | 37.13 | 549.78 | 7556.32 | 787.94 | 36.03 |
| 480 | 37.7 | 37.3 | 37.3 | 37.3 | 37.30 | 37.27 | 552.80 | 7639.70 | 797.85 | 36.48 |
| 490 | 37.8 | 37.5 | 37.4 | 37.5 | 37.47 | 37.35 | 555.79 | 7722.64 | 807.86 | 36.94 |
