# Supplementary material for: Convective heat transfer of the Taylor flow in a two-dimensional piston pump
Source: PLoS One. 2022 Oct 13;17(10):e0275897. doi: 10.1371/journal.pone.0275897 (PMC9560506; doi:10.1371/journal.pone.0275897)
Supplement: S3 Table — (DOCX) [file pone.0275897.s003.docx]

| **S3 Table. The experimental and simulation values with calculated results at 2000 rpm.** | | | | | | | | | | |
| --- | --- | --- | --- | --- | --- | --- | --- | --- | --- | --- |
| ***t*** | $\text{T}_{\text{oil}}$ | $\text{T}_{\text{No.2}}$ | $\text{T}_{\text{No.3}}$ | $\text{T}_{\text{No.4}}$ | $\text{T}_{\text{r2}}$ | $\text{T}_{\text{r2}\text{s}}$ | $\text{R}_{\text{e}}$ | $\text{T}_{\text{a}}$ | $\text{h}_{\text{1}}$ | $\text{N}_{\text{u}\text{1}}$ |
| 0 | 29.2 | 28.5 | 28.2 | 28.3 | 28.33 | 29.00 | 484.85 | 5877.05 | 461.40 | 21.27 |
| 1 | 30.0 | 29.0 | 29.0 | 29.1 | 29.03 | 29.32 | 502.10 | 6302.65 | 474.35 | 21.66 |
| 2 | 30.6 | 29.9 | 29.9 | 29.7 | 29.83 | 29.72 | 519.07 | 6735.95 | 486.33 | 22.06 |
| 3 | 31.1 | 30.4 | 30.4 | 30.4 | 30.40 | 30.18 | 535.77 | 7176.13 | 497.35 | 22.47 |
| 4 | 31.8 | 31.1 | 30.9 | 31.0 | 31.00 | 30.70 | 552.17 | 7622.42 | 507.45 | 22.87 |
| 5 | 32.3 | 31.6 | 31.6 | 31.6 | 31.60 | 31.26 | 568.30 | 8074.14 | 516.72 | 23.28 |
| 6 | 32.9 | 32.2 | 32.2 | 32.1 | 32.17 | 31.82 | 584.15 | 8530.66 | 525.29 | 23.69 |
| 7 | 33.5 | 32.8 | 32.6 | 32.7 | 32.70 | 32.39 | 599.71 | 8991.41 | 533.27 | 24.11 |
| 8 | 33.9 | 33.3 | 33.3 | 33.3 | 33.30 | 32.93 | 615.01 | 9455.91 | 540.83 | 24.52 |
| 9 | 34.4 | 33.9 | 33.6 | 33.7 | 33.73 | 33.43 | 630.04 | 9923.73 | 548.10 | 24.93 |
| 10 | 35.0 | 34.2 | 34.2 | 34.3 | 34.23 | 33.96 | 644.81 | 10394.52 | 555.23 | 25.35 |
| 11 | 35.3 | 34.7 | 34.7 | 34.8 | 34.73 | 34.45 | 659.33 | 10867.96 | 562.36 | 25.76 |
| 12 | 35.8 | 35.1 | 35.3 | 35.1 | 35.17 | 34.90 | 673.61 | 11343.81 | 569.61 | 26.18 |
| 13 | 36.3 | 35.6 | 35.5 | 35.6 | 35.57 | 35.37 | 687.66 | 11821.87 | 577.11 | 26.60 |
| 14 | 36.7 | 36.2 | 36.1 | 36.2 | 36.17 | 35.83 | 701.48 | 12301.97 | 584.94 | 27.02 |
| 15 | 37.1 | 36.6 | 36.4 | 36.5 | 36.50 | 36.27 | 715.09 | 12784.01 | 593.20 | 27.45 |
| 16 | 37.6 | 36.9 | 36.9 | 36.9 | 36.90 | 36.71 | 728.50 | 13267.91 | 601.94 | 27.88 |
| 17 | 37.9 | 37.4 | 37.3 | 37.3 | 37.33 | 37.13 | 741.72 | 13753.61 | 611.21 | 28.31 |
| 18 | 38.3 | 37.7 | 37.6 | 37.6 | 37.63 | 37.52 | 754.75 | 14241.08 | 621.06 | 28.76 |
| 19 | 38.6 | 38.1 | 38.1 | 38.1 | 38.10 | 37.89 | 767.60 | 14730.33 | 631.48 | 29.20 |
| 20 | 39.0 | 38.5 | 38.5 | 38.4 | 38.47 | 38.25 | 780.29 | 15221.37 | 642.49 | 29.66 |
| 21 | 39.4 | 38.8 | 38.7 | 38.8 | 38.77 | 38.63 | 792.82 | 15714.22 | 654.06 | 30.13 |
| 22 | 39.7 | 39.2 | 39.2 | 39.2 | 39.20 | 38.99 | 805.21 | 16208.92 | 666.16 | 30.60 |
| 23 | 40.0 | 39.6 | 39.4 | 39.4 | 39.47 | 39.33 | 817.45 | 16705.50 | 678.73 | 31.09 |
| 24 | 40.4 | 39.9 | 39.8 | 39.9 | 39.87 | 39.67 | 829.55 | 17204.01 | 691.72 | 31.59 |
| 25 | 40.7 | 40.2 | 40.2 | 40.1 | 40.17 | 40.02 | 841.53 | 17704.48 | 705.04 | 32.10 |
| 26 | 41.0 | 40.5 | 40.5 | 40.5 | 40.50 | 40.34 | 853.39 | 18206.95 | 718.60 | 32.62 |
| 27 | 41.4 | 41.0 | 40.7 | 40.8 | 40.83 | 40.68 | 865.13 | 18711.45 | 732.30 | 33.16 |
| 28 | 41.6 | 41.2 | 41.1 | 41.2 | 41.17 | 40.99 | 876.77 | 19218.00 | 746.04 | 33.71 |
| 29 | 41.9 | 41.4 | 41.5 | 41.4 | 41.43 | 41.28 | 888.29 | 19726.60 | 759.72 | 34.28 |
| 30 | 42.3 | 41.7 | 41.7 | 41.7 | 41.70 | 41.60 | 899.72 | 20237.26 | 773.23 | 34.87 |
| 31 | 42.5 | 42.1 | 42.0 | 42.0 | 42.03 | 41.91 | 911.04 | 20749.97 | 786.50 | 35.48 |
| 32 | 42.7 | 42.3 | 42.4 | 42.3 | 42.33 | 42.16 | 922.27 | 21264.68 | 799.45 | 36.10 |
| 33 | 43.1 | 42.7 | 42.6 | 42.7 | 42.67 | 42.44 | 933.41 | 21781.37 | 812.05 | 36.74 |
| 34 | 43.4 | 43.0 | 42.8 | 42.9 | 42.90 | 42.76 | 944.46 | 22299.97 | 824.31 | 37.40 |
| 35 | 43.6 | 43.2 | 43.1 | 43.1 | 43.13 | 43.03 | 955.41 | 22820.42 | 836.27 | 38.08 |
| 36 | 44.0 | 43.4 | 43.5 | 43.3 | 43.40 | 43.33 | 966.28 | 23342.63 | 848.04 | 38.77 |
| 37 | 44.1 | 43.6 | 43.7 | 43.7 | 43.67 | 43.59 | 977.07 | 23866.52 | 859.79 | 39.49 |
| 38 | 44.4 | 44.0 | 43.9 | 43.9 | 43.93 | 43.83 | 987.76 | 24391.97 | 871.78 | 40.22 |
| 39 | 44.7 | 44.4 | 44.2 | 44.2 | 44.27 | 44.10 | 998.38 | 24918.89 | 884.36 | 40.97 |
| 40 | 45.0 | 44.6 | 44.5 | 44.5 | 44.53 | 44.39 | 1008.90 | 25447.14 | 897.96 | 41.74 |
| 41 | 45.2 | 44.8 | 44.7 | 44.7 | 44.73 | 44.65 | 1019.35 | 25976.61 | 913.18 | 42.51 |
| 42 | 45.5 | 45.1 | 45.0 | 45.0 | 45.03 | 44.91 | 1029.70 | 26507.18 | 930.78 | 43.30 |
| 43 | 45.6 | 45.3 | 45.2 | 45.2 | 45.23 | 45.13 | 1039.98 | 27038.73 | 951.72 | 44.10 |
| 44 | 45.9 | 45.6 | 45.4 | 45.5 | 45.50 | 45.35 | 1050.16 | 27571.14 | 977.28 | 44.91 |
| 45 | 46.1 | 45.8 | 45.8 | 45.8 | 45.80 | 45.58 | 1060.27 | 28104.31 | 1009.18 | 45.72 |
| 46 | 46.3 | 46.0 | 45.9 | 45.9 | 45.93 | 45.80 | 1070.29 | 28638.15 | 1049.79 | 46.52 |
| 47 | 46.6 | 46.2 | 46.1 | 46.1 | 46.13 | 46.04 | 1080.23 | 29172.60 | 1102.56 | 47.33 |
