# Supplementary material for: Convective heat transfer of the Taylor flow in a two-dimensional piston pump
Source: PLoS One. 2022 Oct 13;17(10):e0275897. doi: 10.1371/journal.pone.0275897 (PMC9560506; doi:10.1371/journal.pone.0275897)
Supplement: S4 Table — (DOCX) [file pone.0275897.s004.docx]

| **S4 Table. The experimental and simulation values with calculated results at 3000 rpm.** | | | | | | | | | | |
| --- | --- | --- | --- | --- | --- | --- | --- | --- | --- | --- |
| ***t*** | $\text{T}_{\text{oil}}$ | $\text{T}_{\text{No.2}}$ | $\text{T}_{\text{No.3}}$ | $\text{T}_{\text{No.4}}$ | $\text{T}_{\text{r2}}$ | $\text{T}_{\text{r2}\text{s}}$ | $\text{R}_{\text{e}}$ | $\text{T}_{\text{a}}$ | $\text{h}_{\text{1}}$ | $\text{N}_{\text{u}\text{1}}$ |
| 0 | 37.0 | 35.9 | 35.2 | 35.4 | 35.50 | 35.70 | 1089.97 | 29700.87 | 713.24 | 33.11 |
| 10 | 38.1 | 37.0 | 37.0 | 37.2 | 37.07 | 37.05 | 1167.37 | 34069.05 | 736.64 | 34.16 |
| 20 | 39.7 | 38.7 | 38.7 | 38.3 | 38.57 | 38.20 | 1243.06 | 38629.71 | 758.60 | 35.25 |
| 30 | 41.1 | 38.1 | 37.6 | 37.7 | 39.90 | 39.47 | 1316.83 | 43350.82 | 779.24 | 36.36 |
| 40 | 42.0 | 41.1 | 41.1 | 41.2 | 41.13 | 40.70 | 1388.56 | 48202.41 | 798.79 | 37.49 |
| 50 | 43.4 | 42.5 | 42.0 | 42.1 | 42.20 | 41.92 | 1458.18 | 53157.24 | 817.57 | 38.63 |
| 60 | 44.6 | 43.7 | 43.3 | 43.4 | 43.47 | 43.11 | 1525.66 | 58191.25 | 835.93 | 39.78 |
| 70 | 45.3 | 44.5 | 44.5 | 44.6 | 44.53 | 44.11 | 1591.02 | 63283.84 | 854.25 | 40.93 |
| 80 | 46.4 | 45.7 | 45.6 | 45.3 | 45.53 | 45.10 | 1654.30 | 68417.91 | 872.93 | 42.07 |
| 90 | 47.4 | 46.4 | 46.3 | 46.4 | 46.37 | 46.11 | 1715.57 | 73579.80 | 892.29 | 43.20 |
| 100 | 48.1 | 47.5 | 47.4 | 47.2 | 47.37 | 46.96 | 1774.93 | 78759.11 | 912.64 | 44.32 |
| 110 | 49.0 | 48.4 | 48.0 | 48.1 | 48.17 | 47.82 | 1832.47 | 83948.48 | 934.18 | 45.42 |
| 120 | 49.6 | 49.0 | 49.0 | 49.0 | 49.00 | 48.64 | 1888.31 | 89143.27 | 957.07 | 46.50 |
| 130 | 50.6 | 50.0 | 49.6 | 49.6 | 49.73 | 49.46 | 1942.59 | 94341.20 | 981.35 | 47.55 |
| 140 | 51.4 | 50.5 | 50.5 | 50.5 | 50.50 | 50.24 | 1995.42 | 99542.04 | 1006.98 | 48.58 |
| 150 | 51.9 | 51.4 | 51.3 | 51.4 | 51.37 | 50.91 | 2046.92 | 104747.27 | 1033.84 | 49.58 |
| 160 | 52.7 | 52.2 | 51.8 | 51.9 | 51.97 | 51.61 | 2097.23 | 109959.66 | 1061.66 | 50.57 |
| 170 | 53.2 | 52.7 | 52.5 | 52.7 | 52.63 | 52.29 | 2146.47 | 115183.06 | 1090.11 | 51.53 |
| 180 | 54.0 | 53.4 | 53.3 | 53.4 | 53.37 | 52.96 | 2194.74 | 120422.00 | 1118.76 | 52.47 |
| 190 | 54.7 | 54.2 | 53.9 | 53.9 | 54.00 | 53.62 | 2242.15 | 125681.43 | 1147.10 | 53.40 |
| 200 | 55.3 | 54.6 | 54.5 | 54.5 | 54.53 | 54.28 | 2288.81 | 130966.50 | 1174.57 | 54.33 |
| 210 | 55.8 | 55.3 | 55.0 | 55.0 | 55.10 | 54.87 | 2334.80 | 136282.28 | 1200.62 | 55.24 |
| 220 | 56.2 | 55.8 | 55.6 | 55.7 | 55.70 | 55.38 | 2380.20 | 141633.58 | 1224.74 | 56.16 |
| 230 | 56.8 | 56.4 | 56.2 | 56.1 | 56.23 | 55.95 | 2425.08 | 147024.74 | 1246.53 | 57.09 |
| 240 | 57.5 | 57.0 | 56.9 | 56.8 | 56.90 | 56.54 | 2469.49 | 152459.50 | 1265.78 | 58.03 |
| 250 | 58.0 | 57.4 | 57.3 | 57.4 | 57.37 | 57.08 | 2513.49 | 157940.83 | 1282.53 | 58.98 |
| 260 | 58.5 | 58.1 | 57.9 | 58.0 | 58.00 | 57.61 | 2557.11 | 163470.85 | 1297.12 | 59.95 |
| 270 | 59.0 | 58.4 | 58.3 | 58.3 | 58.33 | 58.12 | 2600.39 | 169050.70 | 1310.27 | 60.95 |
| 280 | 59.4 | 59.0 | 58.9 | 58.9 | 58.93 | 58.58 | 2643.34 | 174680.50 | 1323.12 | 61.98 |
| 290 | 59.9 | 59.6 | 59.2 | 59.2 | 59.33 | 59.05 | 2685.96 | 180359.30 | 1337.26 | 63.04 |
| 300 | 60.4 | 59.9 | 59.8 | 59.8 | 59.83 | 59.53 | 2728.26 | 186085.01 | 1354.86 | 64.13 |
| 310 | 60.9 | 60.3 | 60.2 | 60.2 | 60.23 | 60.03 | 2770.23 | 191854.46 | 1378.74 | 65.27 |
| 320 | 61.3 | 61.0 | 60.7 | 60.7 | 60.80 | 60.48 | 2811.86 | 197663.36 | 1412.75 | 66.45 |
| 330 | 61.8 | 61.4 | 61.2 | 61.2 | 61.27 | 60.95 | 2853.11 | 203506.40 | 1462.21 | 67.68 |
| 340 | 62.1 | 61.7 | 61.6 | 61.7 | 61.67 | 61.35 | 2893.97 | 209377.25 | 1535.03 | 68.95 |
| 350 | 62.6 | 62.2 | 62.1 | 61.9 | 62.07 | 61.80 | 2934.41 | 215268.72 | 1643.80 | 70.27 |
