# Supplementary material for: Convective heat transfer of the Taylor flow in a two-dimensional piston pump
Source: PLoS One. 2022 Oct 13;17(10):e0275897. doi: 10.1371/journal.pone.0275897 (PMC9560506; doi:10.1371/journal.pone.0275897)
Supplement: S5 Table — (DOCX) [file pone.0275897.s005.docx]

| **S5 Table. The experimental and simulation values with calculated results at 4000 rpm.** | | | | | | | | | | |
| --- | --- | --- | --- | --- | --- | --- | --- | --- | --- | --- |
| ***t*** | $\text{T}_{\text{oil}}$ | $\text{T}_{\text{No.2}}$ | $\text{T}_{\text{No.3}}$ | $\text{T}_{\text{No.4}}$ | $\text{T}_{\text{r2}}$ | $\text{T}_{\text{r2}\text{s}}$ | $\text{R}_{\text{e}}$ | $\text{T}_{\text{a}}$ | $\text{h}_{\text{1}}$ | $\text{N}_{\text{u}\text{1}}$ |
| 0 | 52.2 | 51.0 | 50.9 | 51.3 | 51.07 | 51.20 | 2651.03 | 175699.24 | 853.28 | 39.29 |
| 10 | 54.1 | 53.0 | 52.3 | 52.5 | 52.60 | 52.89 | 2823.74 | 199337.24 | 874.98 | 40.32 |
| 20 | 55.8 | 54.2 | 54.2 | 54.3 | 54.23 | 54.10 | 2989.48 | 223424.33 | 898.74 | 41.45 |
| 30 | 57.0 | 56.0 | 55.4 | 55.4 | 55.60 | 55.40 | 3148.93 | 247893.88 | 924.60 | 42.67 |
| 40 | 58.1 | 57.2 | 57.0 | 57.2 | 57.13 | 56.63 | 3302.82 | 272716.10 | 952.60 | 43.99 |
| 50 | 59.5 | 58.7 | 58.1 | 58.3 | 58.37 | 57.94 | 3451.90 | 297889.71 | 982.73 | 45.41 |
| 60 | 60.5 | 59.7 | 59.5 | 59.5 | 59.57 | 59.16 | 3596.85 | 323434.01 | 1014.95 | 46.93 |
| 70 | 61.8 | 60.9 | 61.0 | 60.5 | 60.80 | 60.40 | 3738.35 | 349381.87 | 1049.22 | 48.54 |
| 80 | 63.1 | 61.9 | 61.8 | 61.8 | 61.83 | 61.69 | 3876.98 | 375773.74 | 1085.48 | 50.25 |
| 90 | 63.9 | 63.2 | 63.0 | 62.7 | 62.97 | 62.76 | 4013.24 | 402652.74 | 1123.62 | 52.04 |
| 100 | 65.0 | 64.3 | 64.2 | 64.0 | 64.17 | 63.79 | 4147.58 | 430060.83 | 1163.56 | 53.91 |
| 110 | 66.2 | 65.1 | 64.9 | 65.1 | 65.03 | 64.92 | 4280.35 | 458035.84 | 1205.16 | 55.87 |
| 120 | 66.9 | 66.3 | 66.0 | 66.1 | 66.13 | 65.86 | 4411.85 | 486609.41 | 1248.28 | 57.90 |
| 130 | 67.9 | 67.3 | 66.8 | 66.6 | 66.90 | 66.78 | 4542.27 | 515805.60 | 1292.74 | 59.99 |
| 140 | 68.7 | 68.0 | 67.9 | 68.0 | 67.97 | 67.67 | 4671.79 | 545640.17 | 1338.36 | 62.13 |
| 150 | 69.8 | 69.2 | 68.6 | 68.8 | 68.87 | 68.66 | 4800.50 | 576120.35 | 1384.91 | 64.32 |
| 160 | 70.4 | 69.8 | 69.8 | 69.6 | 69.73 | 69.49 | 4928.47 | 607245.09 | 1432.11 | 66.54 |
| 170 | 71.2 | 70.6 | 70.4 | 70.5 | 70.50 | 70.28 | 5055.71 | 639005.63 | 1479.69 | 68.78 |
| 180 | 72.1 | 71.6 | 71.5 | 71.0 | 71.37 | 71.15 | 5182.23 | 671386.50 | 1527.30 | 71.02 |
| 190 | 73.0 | 72.5 | 72.0 | 72.1 | 72.20 | 72.06 | 5307.98 | 704366.57 | 1574.60 | 73.25 |
| 200 | 73.6 | 73.1 | 73.0 | 72.8 | 72.97 | 72.80 | 5432.94 | 737920.47 | 1621.18 | 75.45 |
| 210 | 74.5 | 74.0 | 73.9 | 73.7 | 73.87 | 73.61 | 5557.05 | 772019.93 | 1666.67 | 77.60 |
| 220 | 75.3 | 74.4 | 74.3 | 74.5 | 74.40 | 74.43 | 5680.27 | 806635.37 | 1710.64 | 79.68 |
| 230 | 75.9 | 75.4 | 75.3 | 75.4 | 75.37 | 75.12 | 5802.54 | 841737.27 | 1752.72 | 81.67 |
| 240 | 76.8 | 76.4 | 75.9 | 75.8 | 76.03 | 75.90 | 5923.84 | 877297.63 | 1792.53 | 83.55 |
| 250 | 77.6 | 76.9 | 76.8 | 76.9 | 76.87 | 76.71 | 6044.14 | 913291.22 | 1829.78 | 85.32 |
| 260 | 78.2 | 77.7 | 77.5 | 77.6 | 77.60 | 77.39 | 6163.43 | 949696.70 | 1864.22 | 86.96 |
| 270 | 78.9 | 78.4 | 78.1 | 78.0 | 78.17 | 78.07 | 6281.71 | 986497.46 | 1895.68 | 88.46 |
| 280 | 79.5 | 79.0 | 78.8 | 79.0 | 78.93 | 78.72 | 6399.01 | 1023682.26 | 1924.11 | 89.81 |
| 290 | 80.2 | 79.7 | 79.6 | 79.4 | 79.57 | 79.41 | 6515.35 | 1061245.57 | 1949.54 | 91.03 |
| 300 | 80.9 | 80.6 | 80.0 | 80.1 | 80.23 | 80.14 | 6630.80 | 1099187.56 | 1972.13 | 92.12 |
| 310 | 81.5 | 81.0 | 80.9 | 80.9 | 80.93 | 80.80 | 6745.41 | 1137513.80 | 1992.14 | 93.08 |
| 320 | 82.2 | 81.8 | 81.7 | 81.4 | 81.63 | 81.48 | 6859.26 | 1176234.61 | 2009.90 | 93.94 |
| 330 | 82.8 | 82.5 | 81.9 | 82.1 | 82.17 | 82.06 | 6972.41 | 1215364.09 | 2025.86 | 94.72 |
