# Supplementary material for: Convective heat transfer of the Taylor flow in a two-dimensional piston pump
Source: PLoS One. 2022 Oct 13;17(10):e0275897. doi: 10.1371/journal.pone.0275897 (PMC9560506; doi:10.1371/journal.pone.0275897)
Supplement: S6 Table — (DOCX) [file pone.0275897.s006.docx]

| **S6 Table. The experimental and simulation values with calculated results at 5000 rpm.** | | | | | | | | | | |
| --- | --- | --- | --- | --- | --- | --- | --- | --- | --- | --- |
| ***t*** | $\text{T}_{\text{oil}}$ | $\text{T}_{\text{No.2}}$ | $\text{T}_{\text{No.3}}$ | $\text{T}_{\text{No.4}}$ | $\text{T}_{\text{r2}}$ | $\text{T}_{\text{r2}\text{s}}$ | $\text{R}_{\text{e}}$ | $\text{T}_{\text{a}}$ | $\text{h}_{\text{1}}$ | $\text{N}_{\text{u}\text{1}}$ |
| 0 | 64.9 | 63.6 | 62.4 | 62.4 | 62.80 | 64.00 | 5032.93 | 633258.79 | 1218.86 | 56.45 |
| 5 | 66.0 | 64.8 | 64.8 | 63.7 | 64.43 | 64.67 | 5297.00 | 701455.98 | 1262.97 | 58.54 |
| 10 | 67.2 | 66.0 | 66.0 | 66.1 | 66.03 | 65.63 | 5555.82 | 771678.03 | 1307.47 | 60.65 |
| 15 | 68.2 | 67.1 | 67.1 | 67.2 | 67.13 | 66.67 | 5809.46 | 843745.23 | 1352.07 | 62.76 |
| 20 | 70.2 | 69.2 | 69.2 | 68.2 | 68.87 | 68.10 | 6058.08 | 917509.58 | 1396.49 | 64.87 |
| 25 | 71.2 | 70.2 | 70.3 | 70.3 | 70.27 | 69.45 | 6301.92 | 992853.39 | 1440.44 | 66.95 |
| 30 | 73.0 | 71.2 | 71.2 | 71.2 | 71.20 | 70.92 | 6541.21 | 1069687.24 | 1483.68 | 69.01 |
| 35 | 73.9 | 73.0 | 73.0 | 72.2 | 72.73 | 72.22 | 6776.27 | 1147947.30 | 1525.98 | 71.02 |
| 40 | 74.8 | 73.9 | 73.9 | 73.9 | 73.90 | 73.28 | 7007.40 | 1227592.40 | 1567.16 | 72.98 |
| 45 | 76.4 | 74.8 | 74.7 | 74.7 | 74.73 | 74.53 | 7234.92 | 1308600.89 | 1607.11 | 74.88 |
| 50 | 77.2 | 76.3 | 76.4 | 75.5 | 76.07 | 75.66 | 7459.14 | 1390967.56 | 1645.76 | 76.72 |
| 55 | 78.0 | 77.1 | 77.2 | 77.2 | 77.17 | 76.60 | 7680.37 | 1474700.54 | 1683.07 | 78.50 |
| 60 | 79.5 | 78.0 | 78.0 | 77.9 | 77.97 | 77.75 | 7898.91 | 1559818.34 | 1719.09 | 80.22 |
| 65 | 80.3 | 79.5 | 79.5 | 78.7 | 79.23 | 78.82 | 8115.04 | 1646347.19 | 1753.89 | 81.89 |
| 70 | 81.0 | 80.3 | 80.2 | 80.2 | 80.23 | 79.70 | 8329.03 | 1734318.43 | 1787.59 | 83.50 |
| 75 | 81.7 | 81.0 | 80.9 | 81.1 | 81.00 | 80.48 | 8541.12 | 1823766.25 | 1820.34 | 85.07 |
| 80 | 83.2 | 82.5 | 81.6 | 81.8 | 81.97 | 81.57 | 8751.52 | 1914725.65 | 1852.31 | 86.61 |
| 85 | 83.8 | 83.2 | 83.1 | 82.5 | 82.93 | 82.53 | 8960.43 | 2007230.61 | 1883.68 | 88.12 |
| 90 | 85.2 | 84.5 | 83.8 | 83.7 | 84.00 | 83.62 | 9168.02 | 2101312.54 | 1914.65 | 89.60 |
| 95 | 85.8 | 85.2 | 84.5 | 84.5 | 84.73 | 84.57 | 9374.43 | 2196998.95 | 1945.42 | 91.08 |
| 100 | 86.5 | 85.8 | 85.8 | 85.7 | 85.77 | 85.35 | 9579.80 | 2294312.36 | 1976.18 | 92.56 |
| 105 | 87.8 | 86.6 | 86.4 | 86.4 | 86.47 | 86.36 | 9784.21 | 2393269.46 | 2007.12 | 94.05 |
| 110 | 88.4 | 87.8 | 87.0 | 87.2 | 87.33 | 87.25 | 9987.75 | 2493880.40 | 2038.43 | 95.56 |
| 115 | 89.1 | 88.4 | 88.4 | 87.8 | 88.20 | 88.00 | 10190.48 | 2596148.40 | 2070.27 | 97.09 |
| 120 | 90.3 | 89.1 | 89.1 | 89.1 | 89.10 | 88.95 | 10392.44 | 2700069.46 | 2102.78 | 98.66 |
| 125 | 91.0 | 90.4 | 89.7 | 89.7 | 89.93 | 89.84 | 10593.64 | 2805632.34 | 2136.11 | 100.26 |
| 130 | 91.5 | 91.0 | 90.9 | 90.4 | 90.77 | 90.50 | 10794.11 | 2912818.60 | 2170.38 | 101.91 |
| 135 | 92.1 | 91.6 | 91.5 | 91.5 | 91.53 | 91.12 | 10993.82 | 3021602.99 | 2205.69 | 103.61 |
| 140 | 93.3 | 92.7 | 92.0 | 92.2 | 92.30 | 92.00 | 11192.77 | 3131953.77 | 2242.12 | 105.36 |
| 145 | 93.8 | 93.3 | 92.6 | 92.6 | 92.83 | 92.76 | 11390.93 | 3243833.39 | 2279.75 | 107.17 |
| 150 | 94.4 | 93.8 | 93.8 | 93.7 | 93.77 | 93.40 | 11588.27 | 3357199.12 | 2318.61 | 109.04 |
| 155 | 95.5 | 95.0 | 94.3 | 94.2 | 94.50 | 94.26 | 11784.74 | 3472003.96 | 2358.74 | 110.97 |
| 160 | 96.0 | 95.5 | 95.2 | 95.0 | 95.23 | 94.99 | 11980.31 | 3588197.51 | 2400.14 | 112.96 |
| 165 | 96.5 | 96.1 | 95.7 | 95.9 | 95.90 | 95.58 | 12174.94 | 3705727.00 | 2442.81 | 115.01 |
| 170 | 97.6 | 96.7 | 96.3 | 96.4 | 96.47 | 96.42 | 12368.57 | 3824538.41 | 2486.70 | 117.12 |
| 175 | 98.1 | 97.7 | 96.7 | 96.9 | 97.10 | 97.15 | 12561.17 | 3944577.59 | 2531.77 | 119.28 |
| 180 | 98.6 | 98.3 | 97.9 | 97.6 | 97.93 | 97.73 | 12752.71 | 4065791.43 | 2577.91 | 121.50 |
| 185 | 99.1 | 98.6 | 98.3 | 98.6 | 98.50 | 98.28 | 12943.15 | 4188129.07 | 2625.03 | 123.76 |
| 190 | 100.1 | 99.7 | 98.8 | 99.1 | 99.20 | 99.07 | 13132.47 | 4311543.07 | 2673.00 | 126.06 |
| 195 | 100.5 | 100.2 | 99.9 | 99.5 | 99.87 | 99.69 | 13320.65 | 4435990.54 | 2721.65 | 128.40 |
| 200 | 101.0 | 100.5 | 100.4 | 100.5 | 100.47 | 100.20 | 13507.68 | 4561434.28 | 2770.80 | 130.76 |
| 205 | 102.0 | 101.5 | 100.9 | 101.0 | 101.13 | 100.95 | 13693.57 | 4687843.82 | 2820.24 | 133.14 |
