# Supplementary material for: Convective heat transfer of the Taylor flow in a two-dimensional piston pump
Source: PLoS One. 2022 Oct 13;17(10):e0275897. doi: 10.1371/journal.pone.0275897 (PMC9560506; doi:10.1371/journal.pone.0275897)
Supplement: S7 Table — (DOCX) [file pone.0275897.s007.docx]

| **S7 Table. The experimental and simulation values with calculated results at 6000 rpm.** | | | | | | | | | | |
| --- | --- | --- | --- | --- | --- | --- | --- | --- | --- | --- |
| ***t*** | $\text{T}_{\text{oil}}$ | $\text{T}_{\text{No.2}}$ | $\text{T}_{\text{No.3}}$ | $\text{T}_{\text{No.4}}$ | $\text{T}_{\text{r2}}$ | $\text{T}_{\text{r2}\text{s}}$ | $\text{R}_{\text{e}}$ | $\text{T}_{\text{a}}$ | $\text{h}_{\text{1}}$ | $\text{N}_{\text{u}\text{1}}$ |
| 0 | 73.9 | 72.3 | 70.6 | 71.0 | 71.30 | 72.50 | 7825.18 | 1530835.93 | 1418.37 | 65.97 |
| 5 | 75.5 | 74.1 | 72.4 | 72.8 | 73.10 | 73.69 | 8358.22 | 1746497.14 | 1478.88 | 68.86 |
| 10 | 77.0 | 75.7 | 75.9 | 75.9 | 75.83 | 75.05 | 8883.82 | 1973056.76 | 1539.60 | 71.76 |
| 15 | 80.0 | 77.3 | 77.4 | 77.6 | 77.43 | 77.10 | 9401.79 | 2209843.72 | 1600.06 | 74.66 |
| 20 | 81.4 | 80.2 | 78.7 | 79.1 | 79.33 | 79.08 | 9912.23 | 2456310.00 | 1659.85 | 77.52 |
| 25 | 82.8 | 81.7 | 81.5 | 81.9 | 81.70 | 80.74 | 10415.44 | 2712034.99 | 1718.57 | 80.34 |
| 30 | 85.4 | 83.0 | 82.9 | 83.3 | 83.07 | 82.78 | 10911.87 | 2976723.37 | 1775.95 | 83.10 |
| 35 | 86.7 | 85.7 | 84.3 | 84.7 | 84.90 | 84.63 | 11402.10 | 3250198.18 | 1831.79 | 85.79 |
| 40 | 87.9 | 86.8 | 86.6 | 87.2 | 86.87 | 86.12 | 11886.78 | 3532390.41 | 1886.02 | 88.40 |
| 45 | 90.3 | 88.2 | 88.2 | 88.3 | 88.23 | 87.98 | 12366.61 | 3823326.09 | 1938.69 | 90.94 |
| 50 | 91.4 | 90.5 | 89.4 | 89.5 | 89.80 | 89.62 | 12842.29 | 4123111.91 | 1989.95 | 93.42 |
| 55 | 92.6 | 91.8 | 91.6 | 92.0 | 91.80 | 90.98 | 13314.53 | 4431920.03 | 2040.02 | 95.85 |
| 60 | 93.7 | 92.8 | 92.7 | 93.1 | 92.87 | 92.21 | 13784.01 | 4749972.60 | 2089.24 | 98.23 |
| 65 | 95.9 | 95.1 | 93.9 | 94.2 | 94.40 | 93.89 | 14251.35 | 5077526.33 | 2137.97 | 100.60 |
| 70 | 96.9 | 96.1 | 96.0 | 96.4 | 96.17 | 95.35 | 14717.14 | 5414857.64 | 2186.62 | 102.96 |
| 75 | 98.0 | 97.3 | 94.0 | 97.4 | 96.23 | 96.56 | 15181.89 | 5762248.29 | 2235.61 | 105.34 |
| 80 | 100.1 | 98.3 | 98.0 | 98.5 | 98.27 | 98.20 | 15646.05 | 6119971.92 | 2285.35 | 107.76 |
| 85 | 101.1 | 100.4 | 100.3 | 99.5 | 100.07 | 99.63 | 16109.97 | 6488281.55 | 2336.27 | 110.24 |
| 90 | 103.2 | 101.6 | 101.4 | 101.7 | 101.57 | 101.32 | 16573.95 | 6867398.16 | 2388.72 | 112.79 |
| 95 | 104.2 | 103.5 | 103.4 | 102.7 | 103.20 | 102.75 | 17038.19 | 7257500.40 | 2443.08 | 115.43 |
| 100 | 105.2 | 104.6 | 104.3 | 103.7 | 104.20 | 103.89 | 17502.82 | 7658715.70 | 2499.62 | 118.18 |
| 105 | 106.1 | 105.5 | 105.3 | 105.6 | 105.47 | 104.90 | 17967.87 | 8071112.74 | 2558.60 | 121.05 |
| 110 | 108.0 | 107.5 | 106.2 | 106.5 | 106.73 | 106.37 | 18433.33 | 8494695.53 | 2620.21 | 124.04 |
| 115 | 108.9 | 108.4 | 108.2 | 107.6 | 108.07 | 107.63 | 18899.10 | 8929399.16 | 2684.57 | 127.17 |
| 120 | 109.8 | 109.3 | 109.1 | 109.4 | 109.27 | 108.64 | 19365.01 | 9375087.40 | 2751.73 | 130.44 |
| 125 | 111.6 | 111.2 | 109.8 | 110.3 | 110.43 | 110.07 | 19830.84 | 9831552.26 | 2821.65 | 133.83 |
| 130 | 112.5 | 112.1 | 111.7 | 111.2 | 111.67 | 111.29 | 20296.32 | 10298515.81 | 2894.17 | 137.36 |
| 135 | 113.4 | 112.9 | 112.6 | 112.9 | 112.80 | 112.28 | 20761.15 | 10775634.14 | 2969.03 | 141.00 |
| 140 | 115.1 | 114.5 | 113.6 | 113.8 | 113.97 | 113.66 | 21224.99 | 11262503.85 | 3045.84 | 144.73 |
| 145 | 115.9 | 115.5 | 114.3 | 114.7 | 114.83 | 114.79 | 21687.48 | 11758671.08 | 3124.05 | 148.53 |
| 150 | 116.8 | 116.2 | 115.9 | 115.5 | 115.87 | 115.74 | 22148.27 | 12263643.20 | 3202.95 | 152.37 |
| 155 | 118.4 | 117.0 | 116.8 | 117.3 | 117.03 | 117.06 | 22606.99 | 12776903.19 | 3281.63 | 156.21 |
| 160 | 119.2 | 118.7 | 117.6 | 118.1 | 118.13 | 118.14 | 23063.33 | 13297926.86 | 3359.01 | 159.98 |
| 165 | 120.0 | 119.4 | 119.3 | 119.6 | 119.43 | 119.01 | 23516.98 | 13826202.94 | 3433.83 | 163.63 |
| 170 | 121.6 | 120.4 | 120.2 | 120.3 | 120.30 | 120.31 | 23967.69 | 14361255.89 | 3504.63 | 167.10 |
