# Supplementary material for: Convective heat transfer of the Taylor flow in a two-dimensional piston pump
Source: PLoS One. 2022 Oct 13;17(10):e0275897. doi: 10.1371/journal.pone.0275897 (PMC9560506; doi:10.1371/journal.pone.0275897)
Supplement: Attachment — Submitted filename: Response to Reviewers.docx [file pone.0275897.s008.docx]

**Original Manuscript ID:** **PONE-D-22-16038.**

**Original Article Title: “Convective heat transfer of the Taylor flow in a two-dimensional piston pump”.**

**To:** Editor

**Re:** Response to reviewers.

Dear Editor,

Thank you for allowing a resubmission of our manuscript, with an opportunity to address the reviewers’ comments.

We are uploading (a) our point-by-point response to the comments, (b) an updated manuscript with red text indicating changes, and (c) a clean updated manuscript without red words.

Best regards,

**Revieser:1**

| Comment 1 | The manuscript's English language should be improved by the authors. |
| --- | --- |
| Response | Thank you very much for your recognition of our research and for this valuable suggestion. We have edited and corrected the language usage, spelling, grammar, and forma errors of the entire manuscript thoroughly. |
| Comment 2 | Introduction needs to enrich the readers with recent papers related to your work, which gives the reader a clear visual of the gap that those studies have not address and covered by this study. |
| Response | Thank you very much for your suggestion, we have added more recent literature related to this manuscript. See the introduction section of the manuscript for details. |
| Comment 3 | The results are not enough to defend the importance of work, please add more quantitative outcomes. |
| Response | Thank you very much for your suggestion. We have added a modified relation to the gas-liquid two-phase flow heat transfer in the pump transmission for the accurate calculation of its thermal status, as shown below. |
| Author action | It’s assumed that the effect of oil cavitation to convective heat transfer from the oil to the contact surfaces in the pump cap is consistent. A modified Dittus and Boelter relation $\text{N}_{\text{u}}=\text{0.023}{\text{αR}_{\text{e}}}^{\text{0.8}}{\text{P}_{\text{r}}}^{\text{0.3}}$ is introduced to cope with the unknown convective heat transfer of the vortex flows in the pump transmission, from 1000 rpm to 6000 rpm. The factor $\text{α}$ is set to correct the deviation caused by oil cavitation. And $\text{α}$ is determined through experimental Nusselt number to the Dittus-Boelter relation, as shown in Table 7. Deducing the correct convective heat transfer coefficients is a necessary step for the accurate calculation of the pump transmission’s thermal status. **Table 7. Mean deviation of experimental Nusselt number to the Dittus-Boelter relation and correction factor** $\text{α}$ **at different speed.** **Speed(rpm)** \| **1000** \| **1500** \| **2000** \| **3000** \| **4000** \| **5000** \| **6000**; **Mean deviation** \| 15.49% \| 3.42% \| -21.11% \| -30.63% \| -45.41% \| -48.27% \| -54.31%; **Correction factor** $\text{α}$ \| 1.155 \| 1.034 \| 0.789 \| 0.694 \| 0.546 \| 0.517 \| 0.457 |
| Comment 4 | The reviewer couldn't see the significant objectives of the current work. It would be better if you add a separate section with Key objectives heading. |
| Response | Thank you very much for your suggestion, we have added some background of this study. See the introduction section of the manuscript for details. The convective heat transfer coefficients deduced in this work is a necessary preparation for the accurate calculation of the pump’s heat loss. Besides, the results show that the traditional empirical relations are not suitable when the speed is above 3000rpm. |
| Comment 5 | Why this study is more important and how it could contribute to solving the relevant problem. |
| Response | Thank you very much for your suggestion, we have made the abstract, introduction and conclusions changed. We hope you can understand that the deducing of the convective heat transfer coefficient is a fundamental work. The results will be set in the whole pump’s simulation model as the key parameters. |
| Comment 6 | The methodology part doesn't clear; it may not catch the attraction. Try to make it more concise and briefer. |
| Response | Thank you very much for your suggestion, we have added some details to catch the attraction, shortened the unnecessary discussion, and made some necessary changes to the abstract, introduction, results, and conclusions. |

**Revieser:2**

| Comment 1 | The manuscript presents a series of experimental and numerical studies of flow in an annulus, which the authors attribute to a Taylor-Couette like system. They attempt to study this system to model it as a lumped element in a heat conduction problem. This approach is sound, and the authors provide a series of simulations and experiments for this. Overall the analysis is well done and the correlations are explained. I would just like the authors to be more careful with their English as there is a number of typos like "Taylore" or "existing literatures" in the manuscript which need to be addressed. |
| --- | --- |
| Response | Thank you very much for your affirmation of our research and for pointing out its shortcomings. We have corrected the typos, syntax and forma errors of the whole manuscript. |
